# Cytokine and Adhesion Molecule Expression Induced by Different Strains of *Staphylococcus aureus* in Type 1 Diabetic Rats: Role of Insulin

**DOI:** 10.3389/fimmu.2018.03165

**Published:** 2019-01-17

**Authors:** Paula R. Knox de Souza, Sabrina S. Ferreira, Fernanda P. B. Nunes, Felipe B. Casagrande, Fernando H. G. Tessaro, Mariana C. F. Silva, José Walber Miranda Costa Cruz, Elsa M. Mamizuka, Joilson O. Martins

**Affiliations:** ^1^Laboratory of Immunoendocrinology, Department of Clinical and Toxicological Analyses, School of Pharmaceutical Sciences (FCF), University of São Paulo (USP), São Paulo, Brazil; ^2^Universidade Paulista, São Paulo, Brazil; ^3^Departamento de Bioquímica, Universidade Federal de São Paulo, São Paulo, Brazil; ^4^Laboratory of Microbiology, Department of Clinical and Toxicological Analyses, School of Pharmaceutical Sciences (FCF), University of São Paulo (USP), São Paulo, Brazil

**Keywords:** insulin, *Staphylococcus aureus*, diabetes mellitus, peritonitis, inflammation, cytokines, innate immune response

## Abstract

**Introduction:** *Staphylococcus aureus* may provoke peritonitis and death, especially in immunocompromized individuals such as diabetic patients. We evaluated the role of insulin in *S. aureus*-induced peritoneal infection in diabetic and non-diabetic rats.

**Materials/Methods:** Alloxan-diabetic male Wistar rats and their respective controls received intraperitoneal injections of different strains of *S. aureus* or sterile phosphate-buffered saline. After 3 days of infection, the first set of diabetic and non-diabetic rats received 4 and 1 IU, respectively, of neutral protamine Hagedorn insulin and were analyzed 8 h later. The second set of diabetic and non-diabetic rats received 4 and 1 IU, respectively, of insulin 2 h before intraperitoneal infection and a half dose of insulin at 5 p.m. for the next 2 days and were analyzed 16 h later. The following measurements were performed: (a) number of cells in the peritoneal lavage fluid (PeLF), white blood cell count, and blood glucose; (b) serum insulin and corticosterone; (c) cytokine levels in the PeLF; (d) expression of adhesion molecules in the vascular endothelium; and (e) microbicidal activity.

**Results:** Diabetic rats showed an increased number of polymorphonuclear leukocytes (PMNs) and increased concentrations of CINC-1, IL-4, and IFN-γ in the PeLF after infection with the ATCC 25923 or N315 αHL^+^ strain. The mesenteric expression of PECAM-1 was increased after infection with the N315 HLA^+^ strain. ICAM-1 expression was increased with ATCC infection. Treatment of diabetic rats with a single dose of insulin restored CINC-1 levels in the PeLF for both strains; however, PMN migration, IL-4, and IFN-γ were restored in rats infected with the ATCC strain, whereas the PeLF concentrations of CINC-2, IL-1β, and IL-4 were increased in N315-infected animals. Insulin restored PMN migration and CINC-2 levels in the PeLF in ATCC-infected rats. After multiple treatments with insulin, the levels of IL-1β, IL-6, and IFN-γ were increased in the PeLF of diabetic rats after infection with either strain, and CINC-2 levels were restored in N315-infected animals.

**Conclusion:** These results suggest that insulin distinctively modulates cytokine production or release, PMN leukocyte migration, and adhesion molecule expression during the course of peritonitis induced by different strains of *S. aureus*.

## Introduction

Diabetes mellitus (DM) promotes disruption of the microvascular compartment; this disruption in turn leads to diabetic organ dysfunction, such as nephropathy (1, 2), which represents one of the most important progressive complications associated with DM, as nephropathy may ultimately result in kidney failure (2–4). In such cases, improvement of the patient's quality of life is essential, and one alternative is to perform peritoneal dialysis (PD) (5). *S. aureus*-induced peritonitis is one of the causes of death in patients treated with PD worldwide (6). Dialysis catheters used in PD are a propitious environment for the formation of bacterial biofilms where the bacteria undergo phenotypic changes, forming colonies composed of several layers, which implicates the need for increased concentrations of antibiotics in the catheter to inhibit bacterial proliferation (7, 8).

Strains of *S. aureus* express several virulence factors that potentiate the infection (9). Such factors include binding proteins, enzymes and toxins that allow the bacteria to evade the host immune response (10). The major toxins presented are Panton-Valentine leucocidin (PVL), which exhibits cytolytic or cytotoxic activity depending on the target cell and toxin levels (11, 12), and alpha-haemolysin (αHL). Specifically, PVL destroys polymorphonuclear leukocytes (PMNs) and causes tissue necrosis (13). Strains of *S. aureus* that express PVL and/or αHL include ATCC 25923 (PVL^+^ and αHL^+^), N315 αHL^+^, and MR108 PVL^+^ (14). Therefore, an effective immune response relies on the phagocytic capacity of PMNs to destroy bacteria in the presence of suitable concentrations of pro-inflammatory cytokines (15, 16). In addition, the secretion of other cytokines is essential for bacterial destruction. For instance, IL-1β produced by monocytes stimulated by endotoxins from Gram-positive bacteria amplifies the response to induce the synthesis of additional inflammatory cytokines (17). The expression of adhesion molecules on the vascular endothelium during infection is also primordial because these molecules drive leukocyte migration to the site of infection (18). During peritonitis, leukocyte recruitment into the peritoneum occurs as a consequence of the higher expression of several adhesion molecules, including selectins, integrins, and immunoglobulins on the endothelial or leukocyte surface (19, 20), recruiting leukocytes to the site of infection (18).

Diabetic patients have a higher risk of infections affecting the mucous membranes and soft tissues (21). Moreover, it has been shown that in the lung, diabetic rats show a reduction in tumor necrosis factor (TNF)-α and interleukin (IL)-1β levels and a reduction in intercellular adhesion molecule (ICAM)-1 expression, consequently displaying fewer leukocyte-endothelial interactions. As a result, diabetic animals, in comparison to non-diabetic controls, recruit fewer phagocytes to the site of infection (22). Additionally, the phagocytes of diabetic animals have a reduced phagocytic and microbicide capacity (23). In addition, it has been previously shown that the levels of these inflammatory mediators are normalized after treatment of diabetic animals with insulin (24). Moreover, peritoneal host defense mechanisms are impaired in immunocompromized patients, such as patients on dialysis and with diabetes (25). Therefore, the aims of the present study were to investigate the mechanisms that underlie the effects of insulin and to better understand the integration of the innate immune and endocrine systems.

## Materials and Methods

### Animals

Male Wistar rats weighing 200 ± 20 g at the beginning of the experiments were selected. The animals were housed at 22°C under a 12 h light-dark cycle, and food and water were offered *ad libitum*. This study was carried out following the guidelines of the National Council for the Control of Animal Experimentation (CONCEA) and was randomized; the animals received from the animal house were randomly redistributed in cages at the beginning of the experiment. We split in the animals into two groups; a control groups and a diabetic group, and the main groups were subdivided into smaller groups according to the treatment. The project was approved by the Ethics Committee on Animal Use (CEUA) at the School of Pharmaceutical Sciences (FCF), University of São Paulo, Brazil (protocol number: CEUA/FCF/375). Surgery was performed under ketamine/xylazine anesthesia, and all efforts were made to minimize animal suffering.

### Alloxan-Induced Diabetes

Male Wistar rats were assigned to either a diabetic group (*n* = 50) or a non-diabetic group (*n* = 50). DM was induced by a single intravenous injection of alloxan monohydrate (42 mg/kg) (Sigma Chemical Co, St. Louis, MO, USA) dissolved in physiological saline (0.9% NaCl) (24). Non-diabetic rats were injected with physiological saline. Ten days later, the induction of diabetes was verified by measuring blood glucose concentrations in blood samples from the tail using a blood glucose monitor (Accu-Check Advantage II, Roche Diagnostica, São Paulo, SP, Brazil).

### *S. aureus*-Induced Peritonitis

Three strains of *S. aureus* were used: ATCC 25923 (PVL^+^ and αHL^+^), N315 αHL^+^ and MR108 PVL^+^ (14). These strains were grown aerobically at 37°C in tryptic soy broth (TSB) (Acumedia, USA). After 12 h, these cultures were incubated in fresh TSB (1:100) and stirred for 2 h at 37°C. Cultures were pelleted, washed, and resuspended in phosphate-buffered saline (PBS) (10 mM, pH 7.4 at 4°C) and adjusted to 5 × 10^8^ colony-forming units (CFU)/mL according to the McFarland nephelometric scale (Nefelobac, Probac Brazil, Brazil) (26). One milliliter of bacterial suspension was injected intraperitoneally (i.p.) into diabetic and non-diabetic rats, while non-infected animals received only sterile PBS. Infected animals were monitored for 3 days before being euthanized for the experiment (27).

Animals infected by each strain had the following parameters analyzed: total and differential leukocyte counts from blood, blood glucose determination, and body mass index variation. Peripheral blood samples were collected from the abdominal aorta into EDTA tubes, and a total blood and differential leukocyte count was performed. The total leukocyte count was determined by an automated hematology counter (ABC Vet, ABX Micros 60, Horiba, Japan), while the differential count was performed using smears that were Rosenfeld stained under optical light microscopy (CX31RBSFA, Olympus, Japan). Peripheral blood samples were collected from animal tails to determine glycaemic levels with a glucose monitor (Accu-Chek Active, Roche, Brazil). Body mass variation was measured on the 13th day (10 day period of alloxan-induced diabetes plus 3 days after *S. aureus* peritonitis induction).

### Insulin Treatment

Diabetic and non-diabetic rats were divided into two groups according to the different insulin treatments.

The first set of diabetic and non-diabetic rats received 4 IU and 1 IU, respectively, of neutral protamine Hagedorn (NPH) insulin (Eli Lilly, São Paulo, SP, Brazil) subcutaneously, 2 h before *S. aureus* i.p. injection. The insulin doses were calculated based on previous studies from our group (24). The second set of diabetic and non-diabetic rats received 4 IU and 1 IU, respectively, of NPH insulin subcutaneously, 2 h before *S. aureus* i.p. injection, followed by 2 IU and 0.5 IU at 5 p.m. daily for the next 2 days. Blood samples were collected 16 h after the last NPH dose [adapted from (28)], and blood glucose levels were determined before euthanasia.

### Determination of Serum Corticosterone and Insulin

Peripheral blood samples were collected from the abdominal aorta into a dry tube, and serum samples were stored at −70°C to determine the serum levels of corticosterone and insulin, as per the manufacturer's instructions for the Corticosterone Immunoassay Kit (Cayman Chemical Company, MI, USA) and the Rat Insulin Enzyme Immunoassay Kit (SPI Bio, Massy Cedex, France).

### Peritoneal Lavage, Count Cells, and Enzyme Immunoassay for Cytokines and Chemokines

Animals were anesthetized with ketamine (Ceva Santé Animale, Brazil) (100 mg/kg) and xylazine (Ceva Santé Animale, Brazil) (10 mg/kg) (ratio 10:1). Blood cells were collected via the abdominal aorta. Peritoneal lavage fluid (PeLF) was collected with 10 mL of sterile PBS using a sterile syringe inserted into the peritoneum (26). Cells from the peritoneal cavity were centrifuged at 500 × g and 4°C for 10 min (5810R, Eppendorf, USA). The supernatant was removed, and the cells were resuspended in 1 mL of PBS to count the total number of cells in the peritoneal cavity. The cell suspension was diluted at a ratio of 1:20 (v:v) in Turk liquid, and the total cell count was determined by optical microscopy using Neubauer chambers (Improved Bright-line, Superior Marienfeld, Germany). Furthermore, by using a cytocentrifuge (SPIN IV Incibras, Brazil), differential cell counting was performed using Rosenfeld-stained smears and optical light microscopy (CX31RBSFA, Olympus, Japan). The concentrations of cytokines [IL-1β, IL-4, IL-6, TNF-α, interferon (IFN)-γ, IL-10, and IL-12] and cytokine-induced neutrophil chemoattractant (CINC-1, CINC-2, and CINC-3) in the PeLF were determined by enzyme-linked immunosorbent assays (ELISA) using commercially available kits, according to the manufacturer's instructions (Duo-set, R & D System, Inc., Minneapolis, MN, USA).

### Isolation of the Mesentery and Adhesion Molecule Expression by Immunohistochemistry

P-selectin, ICAM, and platelet endothelial cell adhesion molecule (PECAM)-1 expression on mesentery microvessels was determined. After anesthesia, a median laparotomy was performed. The mesentery was gently removed and immersed in hexane and frozen in liquid nitrogen. Serial 5 μm cryostat sections were obtained from frozen fragments of mesentery and placed directly on silanized slides. After fixation in acetone, sections were incubated with hydrogen peroxide at 1% for approximately 30 min at room temperature to block endogenous peroxidase. The tissue samples were incubated with primary antibodies (anti-P-selectin, anti-ICAM-1, and anti-PECAM-1) (Santa Cruz Biotechnology, Dallas, Texas, USA) overnight in a humid chamber at 4°C (Santa Cruz Biotechnology). After washing the slides with PBS, samples were incubated with a secondary antibody (Anti-goat IgG Horseradish Peroxidase-conjugated) (Sigma Aldrich Company). One hour later, the reaction was developed with a solution containing Tris-HCl (0.1 M pH 7.6), hydrogen peroxide 30% and 3.3 diaminobenzidine (DAB, Sigma Aldrich Company). After processing, the sections were counterstained in Harris haematoxylin, dehydrated, and mounted in Entellan synthetic resin (Merck). Slides were observed under a light microscope (Nikon Eclipse 80i, Tokyo, Japan) and photographed using the NIS-Elements AR 3.1 (SP3 build 634) imaging software (Nikon). The positive area was measured (μm^2^), and the maximum number of mesentery microvessels per slide was determined. Vessel diameters were standardized to rule out the influence of the vessel gauge. The results are expressed as the mean total/diameter of the area for each animal [NIS-Elements AR 3.1 (SP3 build 634) imaging software-Nikon].

### Isolation of Peritoneal Macrophages and Determination of Microbicide Activity

Peritoneal macrophages (PMs) were obtained by peritoneal lavage as described above. PeLF was centrifuged at 259 × g for 10 min and suspended in RPMI-1640 medium. The cell suspension was adjusted and distributed to 5 × 10^5^ cells per well. Then, the cells were placed in 96-well plates and incubated at 37°C and 5% CO_2_ for 2 h to adhere. Next, the cells were washed three times using pre-warmed PBS and incubated for an additional 12 h in RPMI-1640 medium containing fetal calf serum (10%). The ability of bacteria to survive among the PMs was quantified using a tetrazolium dye reduction assay (29). MP were infected with a 0.1-mL suspension of *S. aureus* (1 × 10^7^ CFU/mL; multiplicity of infection [MOI], 50:1) for 30 min in PBS to allow phagocytosis to occur. The intensity of the absorbance at 595 nm was directly proportional to the number of intracellular bacteria associated with the macrophages; the protocol for bacterial death was performed as previously described (29).

### Statistical Analysis

Comparisons between two groups, such as the standardization of the experimental model and DM, were evaluated using the *t*-test. The following data distribution applies to control (non-diabetic) and diabetic animals 10 days after alloxan injection. In the follow-up graphs with comparisons of 8 groups, the two-way ANOVA model was used, and when significant differences were identified, individual comparisons were later conducted with the Bonferroni *t*-test for unpaired values. All tests were performed using GraphPad Prism (version 6.0 for Windows, GraphPad Software, La Jolla, CA, USA). A two-tailed *p*-value with 95% confidence interval was defined. Data are presented as the mean ± standard error of the mean (SEM). *P*-values < 0.05 were considered significant.

## Results

### Experimental Model of Diabetes Mellitus and Peritoneal Inflammation

**Figure 1:**
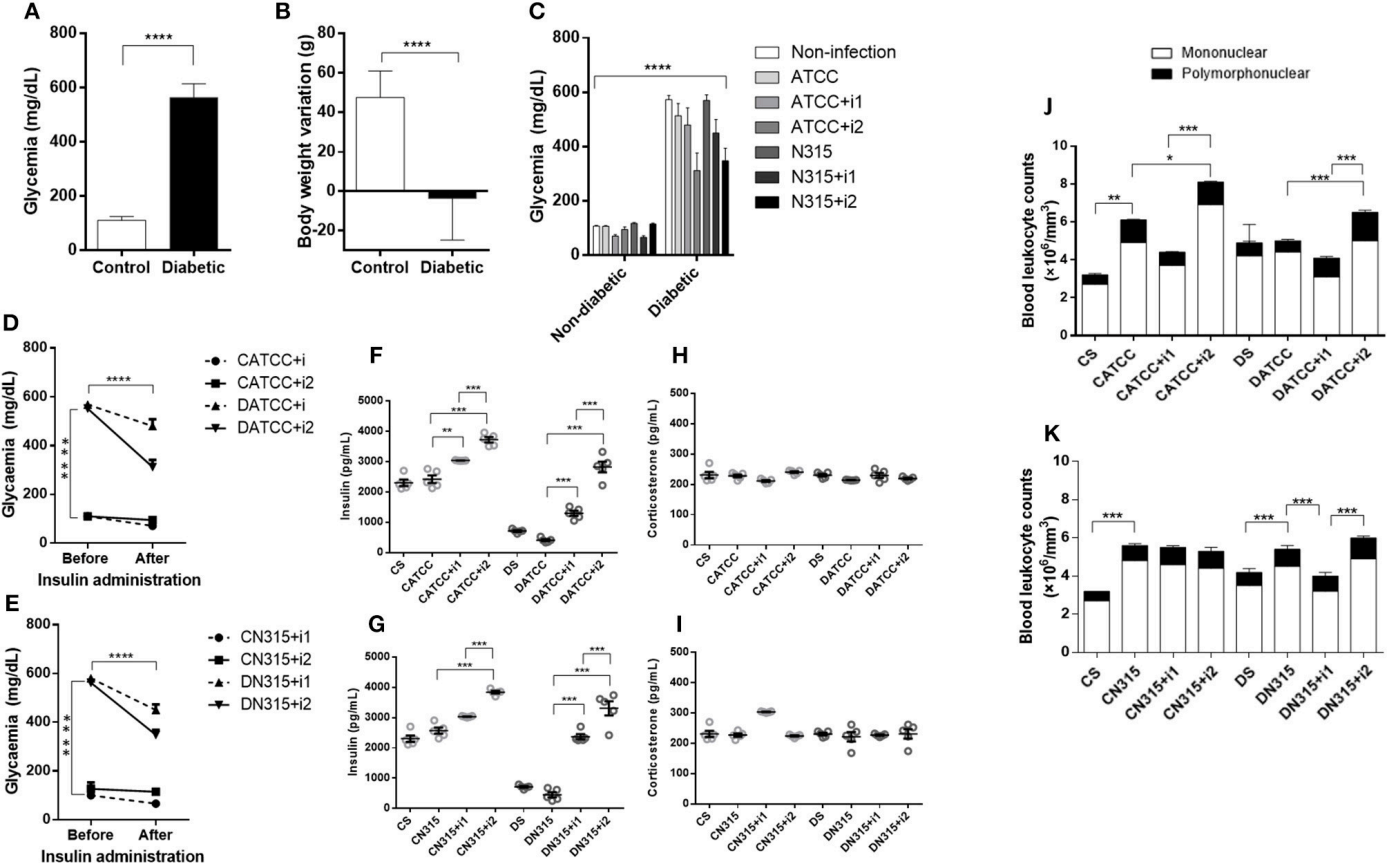
DM characterization. DM was induced by alloxan injection (42 mg/kg, i.v.) 10 days before infection with *S. aureus* strains, and measurements were carried out 3 days afterward (13 days total). **(A)** Blood glucose (mg/dL) 10 days after alloxan injection, *n* = 49; **(B)** Body weight variation (g) 10 days after alloxan injection, *n* = 49. **(C)** Blood glucose (mg/dL) 13 days after alloxan injection, *n* = 7. **(D)** Effect of insulin treatment on glycaemia in diabetic and non-diabetic rats infected with *S. aureus* strain ATCC 25923, *n* = 5. **(E)** Effect of insulin treatment on glycaemia in diabetic and non-diabetic rats infected with *S. aureus* strain N315 αHL^+^, *n* = 5. **(F)** Effect of insulin treatment on insulin concentrations in diabetic animals infected with *S. aureus* strain ATCC 25923, *n* = 5. **(G)** Effect of insulin treatment on insulin concentrations in diabetic animals infected with *S. aureus* strain N315 αHL^+^, *n* = 5. **(H)** Effect of insulin treatment on serum corticosterone concentrations in diabetic animals infected with *S. aureus* strain ATCC 25923, *n* = 5. **(I)** Effect of insulin treatment on serum corticosterone concentrations in diabetic animals infected with S. aureus strain N315 αHL^+^, *n* = 5. **(J)** Blood leukocyte counts after infection with S. aureus strain ATCC 25923, *n* = 7 **(K)** Blood leukocyte counts after infection with *S. aureus* strain N315 αHL^+^. *n* = 7. Values represent the mean ± SEM. ^*^p < 0.05; ^**^p < 0.01, ^***^p < 0.001, and ^****^p < 0.001. Differences among the initial groups (diabetic or not) were analyzed using Student's *t*-test. Differences among the groups were tested with two-way analysis of variance followed by Bonferroni *post hoc* test. (GraphPad Prism version 6.0 for Windows, GraphPad Software, La Jolla, CA, USA).

Experiments were performed using male Wistar rats. After 10 days of intravenous alloxan administration (42 mg/kg), the animals became diabetic (blood glucose level >200 mg/dL) and exhibited body weight reduction compared to the non-diabetic group, which showed normoglycaemia (Figure 1A) and higher weight gain in the same period (Figure 1B). Peritonitis was induced in both groups. Initially, three different *S. aureus* strains were used to induce peritonitis: ATCC 25923, N315 HLA+, and MR108 PVL+. Three days after peritonitis induction, MR108 PVL+ caused intense peritoneal coagulation in 12 animals, making it impossible to collect PeLF. Moreover, 12 animals were used during standardization; 6 diabetic animals that received the treatment died (100% of the diabetic rats), while the 6 animals in the control group survived but presented coagulation of the peritoneal lavage fluid, as noted, making it impossible to obtain lavage samples for quantification of chemokines and cytokines. For this reason, this strain was removed from the experiments. Analyses were performed 3 days after intraperitoneal injection of *S. aureus* ATCC 25923 (ATCC) and N315 HLA+ (N315) strains. Based on these results, we can conclude that the peritonitis model worked and did not cause severe peritonitis, since there was 100% survival.

### Effects of ATCC 25923 Infection

#### Blood Glucose After Insulin Treatment and Serum Insulin and Corticosterone Levels

Blood glucose levels were similar before and after insulin treatments in non-diabetic animals (Figure 1C). In contrast, blood glucose levels in diabetic animals decreased after treatment with multiple doses of insulin (Figures 1D,E), albeit not to the control blood glucose levels. The insulin serum concentration in diabetic animals was drastically lower than that in non-diabetic animals. Treatment of diabetic animals with a single dose of insulin (D_ATCC+i1_) resulted in a 2-fold increase, whereas treatment with multiple doses (D_ATCC+i2_) promoted a 4-fold increase in the serum concentration of insulin (*S. aureus*) (Figure 1F). ATCC 25923 infection did not alter the corticosterone levels under diabetic conditions or after insulin treatments (Figure 1H).

#### Cytokine Secretion Induced by Strain ATCC 25923 of *S. aureus*

**Figure 2:**
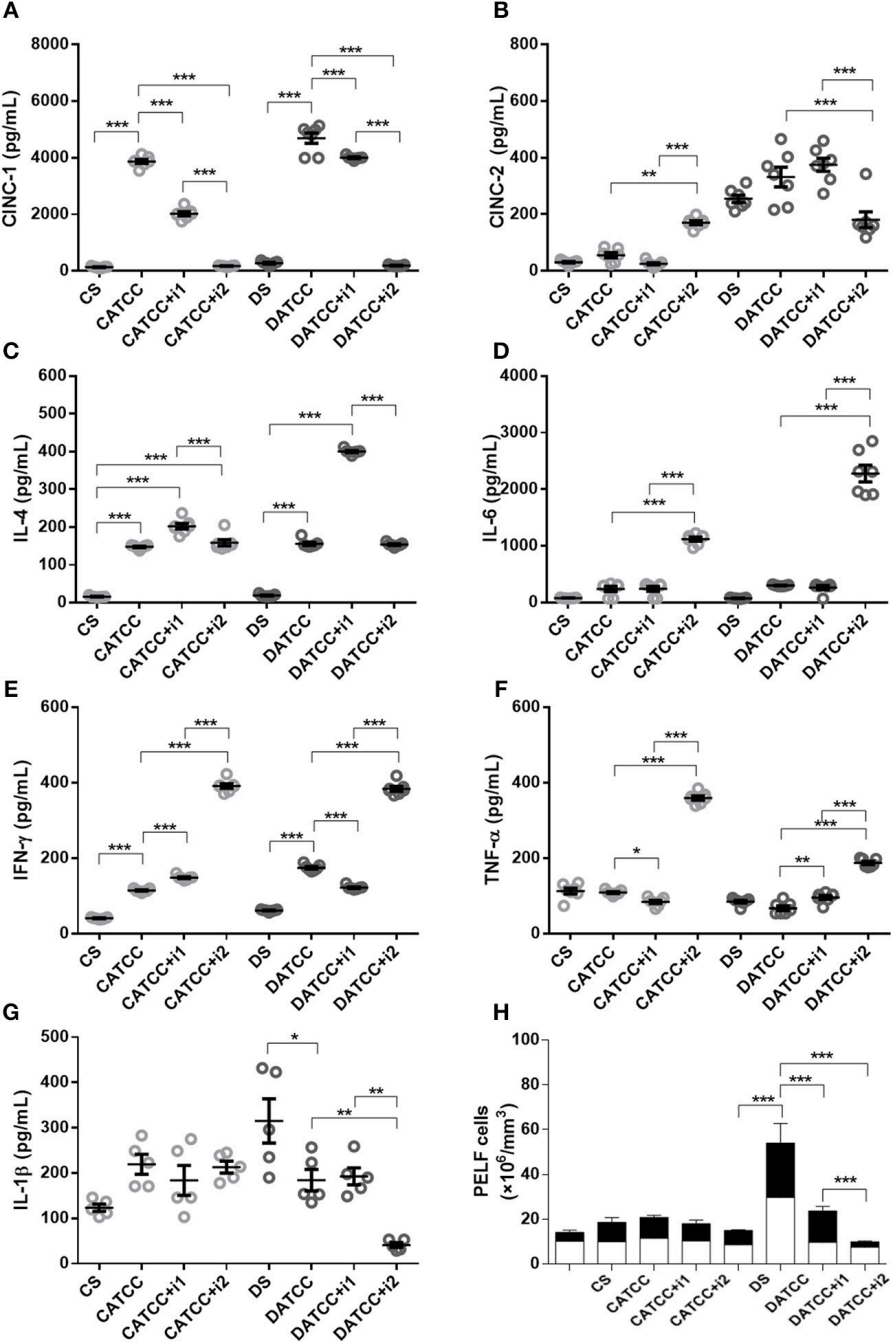
Effect of insulin on PeLF cytokine concentrations after *S. aureus* strain ATCC 25923 infection. **(A)** CINC-1, **(B)** CINC-2, **(C)** IL-4, **(D)** IL-6, **(E)** IFN-γ, **(F)** TNF-α, and **(G)** IL-1β PeLF concentrations **(H)** Blood leucocyte count in PelF. PeLF was analyzed 3 days after *S. aureus* strain ATCC 25923 infection. Animals were treated with single-dose insulin (I1) or with multiple doses of insulin (I2). Non-diabetic uninfected group (Cs); Non-diabetic infected group (CATCC); Non-diabetic infected; and treated with I1 insulin group (CATCC+I1); Non-diabetic infected and treated with I2 insulin group (CATCC+I2); Diabetic uninfected group (Ds); Diabetic infected group (DATCC); Diabetic infected and treated with I1 insulin group (DATCC+I1); Diabetic infected and treated with I2 insulin group (DATCC+I2). Values are shown as the mean ± SEM of seven animals/group. ^*^p < 0.05; ^**^p < 0.01, ^***^p < 0.001. Differences among the groups were tested with two-way analysis of variance followed by Bonferroni *post hoc* test. (GraphPad Prism version 6.0 for Windows, GraphPad Software, La Jolla, CA, USA).

We determined the concentration of pro-inflammatory (IL1β, IL-6, TNF-α, IFNγ, IL-12), chemokine (CINC-1, CINC-2 e CINC-3) and anti-inflammatory (IL-4 e IL-10) cytokines in PeLF supernatants. Infected non-diabetic animals (C_ATCC_) had 28.5-fold higher CINC-1 levels than non-infected non-diabetic controls (Cs). A single dose of insulin (C_ATCC+i1_) reduced the level of CINC-1 by almost 48%, while treatment with multiple doses of insulin (C_ATCC+i2_) diminished the effect by 95%. Diabetic rats (D_ATCC_) presented an increased production of CINC-1, while insulin treatment diminished the CINC-1 secretion by approximately 16.6% (D_ATCC+i1_) and 96% (D_ATCC+i2_) (Figure 2A). The production of CINC-2 by diabetic rats was higher than the production of CINC-2 by control animals, regardless of the presence of bacteria or the type of insulin treatment performed. The production of CINC-2 by the uninfected diabetic group (DS) was ~8.3 times higher than in the control group (CS), and the infection of diabetic animals (DATCC) with the bacterium increased CINC-2 production by approximately 6 times in relation to the control group (CATCC). Treatment with one dose of insulin (DATCC+i1) did not alter the production of CINC-2, but treatment with four doses of insulin (DATCC+i2) decreased CINC-2 production by 45% in relation to infected diabetic animals (DATCC). Nevertheless, the four dose treatment (DATCC+i2) reduced CINC-2 production by 52% compared with the single-dose treatment (DATCC+i1) (Figure 2B). The IL-4 levels were 9.3-fold higher in the C_ATCC_ group than in the Cs group. A single dose (C_ATCC+i1_), but not multiple doses (C_ATCC+i2_), of insulin led to a further 37% increase in IL-4. Infection had a similar effect on diabetic animals (D_ATCC_), inducing an 8-fold increase in the IL-4 level in comparison with that of uninfected diabetic controls (Ds). However, in diabetic animals, a single dose of insulin (D_ATCC+i1_) had a markedly stronger effect than that in non-diabetic rats, namely, a 2.6-fold increase in IL-4 concentrations (Figure 2C). Infection with the ATCC 25923 strain (C_ATCC_ and D_ATCC_) did not alter IL-6 levels, but treatment with multiple doses of insulin increased IL-6 production (7- to 6-fold) in the PeLF from infected diabetic rats (D_ATCC+i2_) compared with that of non-insulin-treated animals (D_ATCC_) (Figure 2D). A single dose of insulin did not alter IFN-γ production, while treatment with multiple doses increased this production by almost 9.6-fold. Diabetic infected rats (D_ATCC_) presented an increase in PeLF IFN-γ levels (2.8-fold) compared with that in Ds rats. This cytokine production was modified by insulin treatment; a single dose (D_ATCC+i1_) diminished the production by almost 30%, while multiple doses (D_ATCC+i2_) increased the production by 2.2-fold (Figure 2E). In both non-diabetic and diabetic infected rats, treatment with multiple doses of insulin increased the levels (3.3- and 2.7-fold, respectively) of TNF-α in the PeLF (Figure 2F). Moreover, infection did not significantly alter IL-1β levels in the PeLF of non-diabetic animals, but it did promote an increase in PeLF IL-1β in diabetic animals (D_ATCC_) (Figure 2G). Treatment with multiple doses of insulin further increased the IL-1β level by 42% (Figure 2G). Finally, PeLF levels of CINC-3, IL-10, and IL-12 were similar across all experimental groups.

#### Peritonitis Caused by Strain ATCC 25923 of *S. aureus*

Infection with the *S. aureus* strain ATCC 25923 increased the total leukocyte (1.8-fold) and neutrophil (2.4-fold) blood counts in non-diabetic animals (CATCC) compared to those of the non-diabetic uninfected control group (Cs). Blood leukocyte counts were equivalent among diabetic groups. Treatment of diabetic animals with a single dose of insulin (DATCC+i1) decreases both total (1.4-fold) and neutrophils (1.6-fold) leukocyte blood counts, whereas treatment with multiple doses (DATCC+i2) promoted an increased leukocytes (1.1-fold) and neutrophil (2.5-fold) numbers in the blood (Figure 1J). However, PeLF leukocyte counts in C_ATCC_ animals remained similar to those in Cs animals. Diabetes potentiated the recruitment of leukocytes induced by infection with the ATCC 25923 strain. Thus, the number of leukocytes in the PeLF was 2- to 3-fold larger in diabetic (D_ATCC_) than in non-diabetic rats (C_ATCC_). The numbers of mononuclear and polymorphonuclear leukocytes in the PeLF did not differ statistically between C_ATCC_ and Cs animals. Among diabetic animals, those infected by the ATCC 25923 strain (D_ATCC_) had 3.6-fold more mono- and polymorphonuclear leukocytes than did non-infected animals (Ds). Treatment with a single dose of insulin (D_ATCC+i1_) decreased the number of leukocytes in the PeLF by 56% compared to those in non-insulin-treated animals (D_ATCC_), whereas with multiple doses of insulin (D_ATCC+i2_), the reduction reached 82%. These results are summarized in Figure 2H.

#### Adhesion Molecule Expression Induced by Strain ATCC 25923 of *S. aureus*

**Figure 3:**
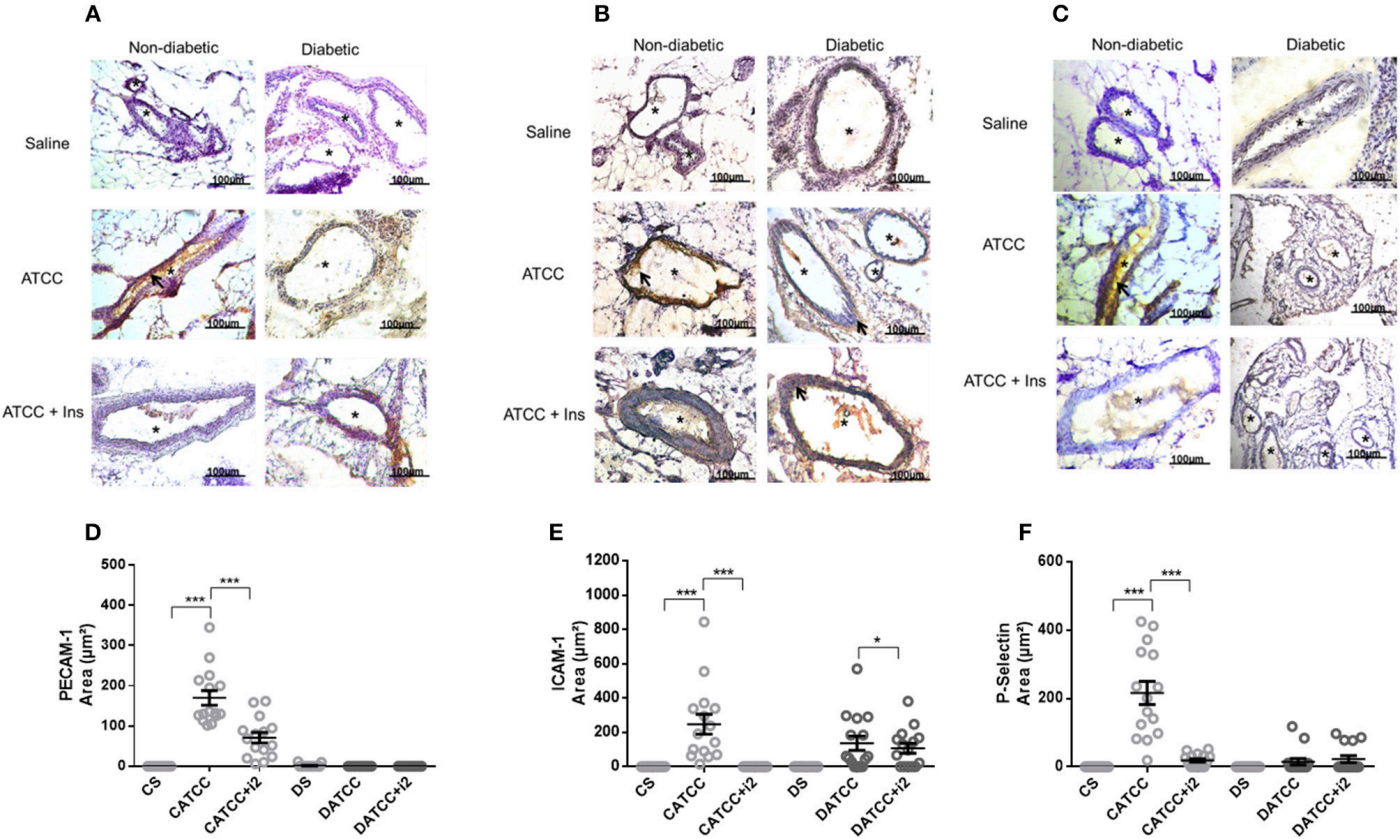
Mesenteric adhesion molecule expression after *S. aureus* strain ATCC 25923 infection: role of insulin. Samples were obtained from the mesentery of diabetic or non-diabetic animals treated or not treated with insulin 3 days after *S. aureus* ATCC 25923 infection and stained with haematoxylin (purple staining) and antibodies (brown staining, arrows) for PECAM **(A)**, ICAM-1 **(B)**, and P-selectin **(C)**. ^*^blood vessel; bar = 50 μm. Quantification of immunostaining was performed with the software NIS-Elements AR. Values represent the mean total/diameter of the area ± s.e.m. of three animals/group (five fields were photographed per animal). **(D)** PECAM; **(E)** ICAM-1; **(F)** P-selectin expression (μm^2^). Animals were treated with multiple doses of insulin (I2). Non-diabetic uninfected group (Cs); Non-diabetic infected group (CATCC); Non-diabetic infected and treated with I2 insulin group (CATCC+I2); Diabetic uninfected group (Ds); Diabetic infected group (DATCC); Diabetic infected; and treated with I2 insulin group (DATCC+I2). Values are shown as the mean ± SEM of 5 animals/group ^***^p < 0.001. Differences among the groups were tested with two-way analysis of variance followed by Bonferroni *post hoc* test (GraphPad Prism version 6.0 for Windows, GraphPad Software, La Jolla, CA, USA).

Non-diabetic animals infected with the ATCC 25923 strain (C_ATCC_) expressed PECAM-1 on the mesenteric vascular endothelium 170-fold more intensely than did uninfected controls (Cs), according to the quantitative evaluation of immunostained samples (Figure 3D). Treatment with multiple doses of insulin (C_ATCC+i2_) countered this effect on PECAM-1. In the infected diabetic animals, PECAM-1 expression could not be detected, and treatment with multiple doses of insulin did not alter this lack of response (Figure 3A). Likewise, the expression of ICAM-1 (Figures 3B,E) and P-selectin (Figures 3C,F) increased in infected non-diabetic animals by 247- and 210-fold, respectively. Treatment with multiple doses of insulin partly reversed the effect on P-selectin (91%) and reduced the ICAM-1 expression to undetectable levels. In diabetic rats (D_ATCC_), infection resulted in the expression of ICAM-1 and P-selectin, but not of PECAM-1, and insulin treatment did not alter this pattern (Figures 3A–F).

#### Microbicide Activity of Peritoneal Macrophages

The microbicide activity of PMs isolated from ATCC 25923-infected animals indicated that insulin treatment did not alter the capacity of macrophages to eliminate bacteria (mean ± SEM; CATCC, 47.6 ± 2.8; CATCC+i2, 51.2 ± 2.6; DATCC, 51.6 ± 4.9; DATCC+i2, 55.9 ± 5.1). These results are expressed as the percent survival.

### Effects of N315 αHL^+^ Infection

#### N315 αHL^+^ Infection: Effects of Insulin Treatment on Blood Glucose, Serum Insulin, and Corticosterone Levels

In non-diabetic animals, blood glucose levels were not significantly reduced by single-dose or multiple-dose treatments with insulin, whereas in diabetic animals, both treatments with insulin reduced the levels of blood glucose, but not enough to bring the animals to normoglycaemia (Figure 1E).

Infected non-diabetic animals treated with a single dose of insulin had 1.2-fold higher serum levels of the hormone than control rats had, whereas in animals treated with multiple doses of insulin, the increase corresponded to 1.5-fold. Infected diabetic animals treated with a single dose or with multiple doses of insulin had 5.2- and 7.3-fold higher levels of the hormone in serum, respectively (Figure 1G). There were no significant differences in corticosterone levels between the different groups studied (Figure 1I).

#### N315 αHL^+^-Induced Cytokine Secretion

**Figure 4:**
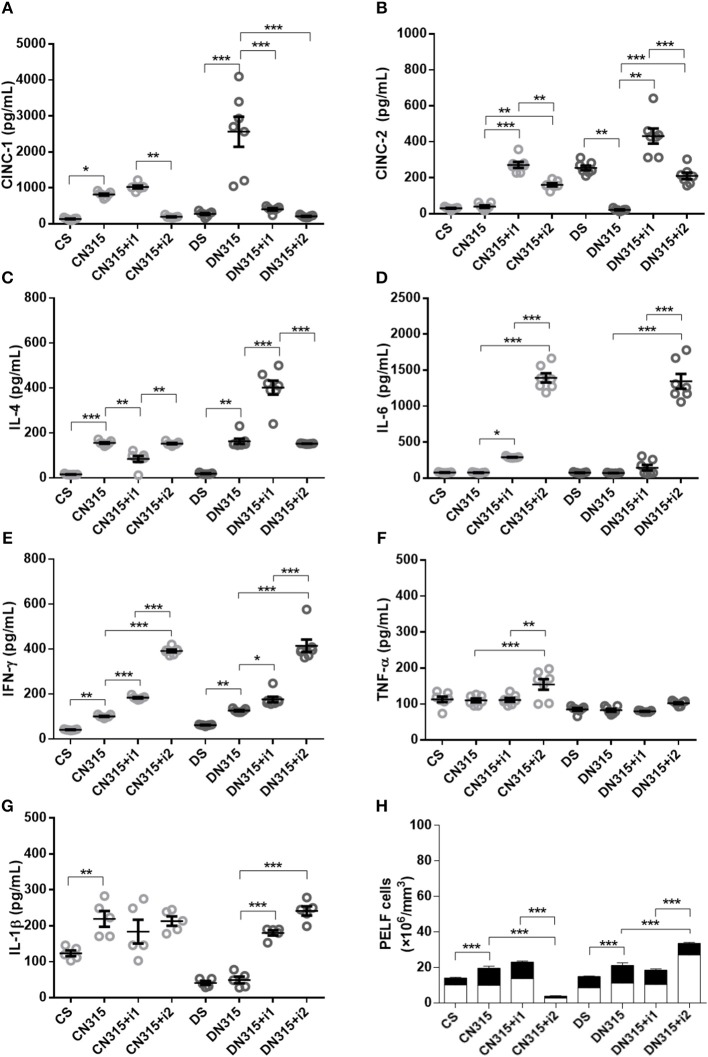
Effect of insulin on PeLF cytokine concentrations after *S. aureus* strain N315 αHL^+^ infection. **(A)** CINC-1, **(B)** CINC-2, **(C)** IL-4, **(D)** IL-6, **(E)** IFN-γ, **(F)** TNF-α, and **(G)** IL-1β PeLF concentrations **(H)** Blood leucocyte count in PelF. PeLF was analyzed 3 days after *S. aureus* strain N315 αHL^+^ infection. Animals were treated with single-dose insulin (I1) or with multiple doses of insulin (I2). Non-diabetic uninfected group (Cs); Non-diabetic infected group (CN315); Non-diabetic infected; and treated with I1 insulin group (CN315+I1); Non-diabetic infected and treated with I2 insulin group (CN315+I2); Diabetic uninfected group (Ds); Diabetic infected group (DN315); Diabetic infected and treated with I1 insulin group (DN315+I1); Diabetic infected; and treated with I2 insulin group (DN315+I2). Values are shown as the mean ± SEM of five animals/group. ^*^p < 0.05; ^**^p < 0.01, ^***^p < 0.001. Differences among the groups were tested with two-way analysis of variance followed by Bonferroni *post hoc* test. (GraphPad Prism version 6.0 for Windows, GraphPad Software, La Jolla, CA, USA).

Infection elevated the concentrations of PeLF CINC-1 in non-diabetic (C_N315_) and diabetic animals (D_N315_). Treatment with multiple doses of insulin reversed this effect by 76% (C_N315+i2_) and 92% (D_N315+i2_), respectively (Figure 4A).

No change in the levels of CINC-2 was observed in infected non-diabetic animals (C_N315_). A single dose of insulin (C_N315+i1_) induced an increase in CINC-2 levels (6.8-fold), whereas treatment with multiple doses (C_N315+i2_) led to a four-fold increase. In addition, diabetic, uninfected animals (Ds) had 8.3-fold higher levels of CINC-2 than did non-diabetic, uninfected animals (C_S_). Meanwhile, bacterial infection (D_N315_) decreased the secretion of CINC-2 by approximately 92%, whereas insulin treatment of infected diabetic animals increased the PeLF CINC-2 by 20.3-fold (D_N315+i1_) and 10-fold (_DN315+i2_) (Figure 4B). Infection increased IL-4 secretion in the PeLF by 9.8-fold in non-diabetic (C_N315_) and 8.6-fold in diabetic (D_N315_) animals. Treatment with a single dose of insulin (C_N315+i1_) was more efficient at modulating cytokine production than was treatment with multiple doses of insulin (Figure 4C). Levels of IL-6 were not altered by infection with N315 αHL^+^. Treatment with multiple doses of insulin induced a sharp 18-fold increase in the IL-6 levels in infected non-diabetic and diabetic animals (C_N315+i2_ and D_N315+i2_) (Figure 4D). Non-diabetic infected animals presented an increase in IFN-γ levels compared to uninfected controls. Both insulin treatments (C_N315+i1_ or C_N315+i2_) were efficient in potentializing the production of this cytokine. In diabetic animals, the infection (DN315) doubled the IFN-γ levels, and treatment with multiple doses of insulin potentiated this effect (3.3-fold) (Figure 4E). Levels of TNF-α were not altered by N315 αHL^+^ infection in either non-diabetic (C_N315_) or diabetic (D_N315_) animals. Multiple doses of insulin increased the TNF-α levels by 38% in non-diabetic rats but had no such effect on diabetic animals (Figure 4F). In diabetic rats, treatment with a single dose of insulin (D_N315+i1_) led to a 3.7-fold increase in IL-1β, whereas with multiple doses (D_N315+i2_), there was a 4.9-fold elevation (Figure 4G). The PeLF concentrations of CINC-3, IL-10, and IL-12 did not vary significantly among experimental groups (data not shown).

#### Peritonitis Caused by Strain N315 αHL^+^

Infection with the *S. aureus* strain N315 aHL^+^ increased the total leukocyte (1.7-fold) and neutrophil (1.6-fold) blood counts in non-diabetic animals (C_N315_) compared to those of the non-diabetic uninfected control group (Cs). Blood leukocyte counts were equivalent among diabetic groups. Treatment of diabetic animals with a single dose of insulin (D_N315+i1_) decreases both total (1.4-fold) and neutrophils (1.1-fold) leukocyte blood counts, whereas treatment with multiple doses (D_N315+i2_) promoted an increased leukocytes (1-fold) and neutrophil (1-fold) numbers in the blood (Figure 1K). In contrast, in non-diabetic animals, the PeLF from infected animals (C_N315_) had 1.4-fold more leukocytes than did the PeLF from non-infected animals (C_S_), while the treatment with multiple doses of insulin (C_N315+i2_) reduced the number of leukocytes in the PeLF by 68%. In diabetic animals, infected animals (D_N315_) also had two-fold more leukocytes in the PeLF than non-infected animals (Ds) had. However, insulin treatment (D_N315+i2_) potentiated the effects of infection, increasing the PeLF leukocytes by 66%. Treatment with multiple doses of insulin increased mononuclear cell migration by 2-fold in diabetic rats (D_N315+i2_) compared to the migration observed in untreated diabetic rats (D_N315_). Multiple doses of insulin generated a 2.5-fold greater cell migration than did a single dose of the hormone (D_N315+i1_). Infection of non-diabetic animals increased the number of PMNs in the peritoneal cavity by 2.5-fold compared with that of control animals (C_S_). In diabetic animals, no alterations to PMN migration were observed (Figure 4H).

#### N315 αHL^+^-Induced Expression of Adhesion Molecules

**Figure 5:**
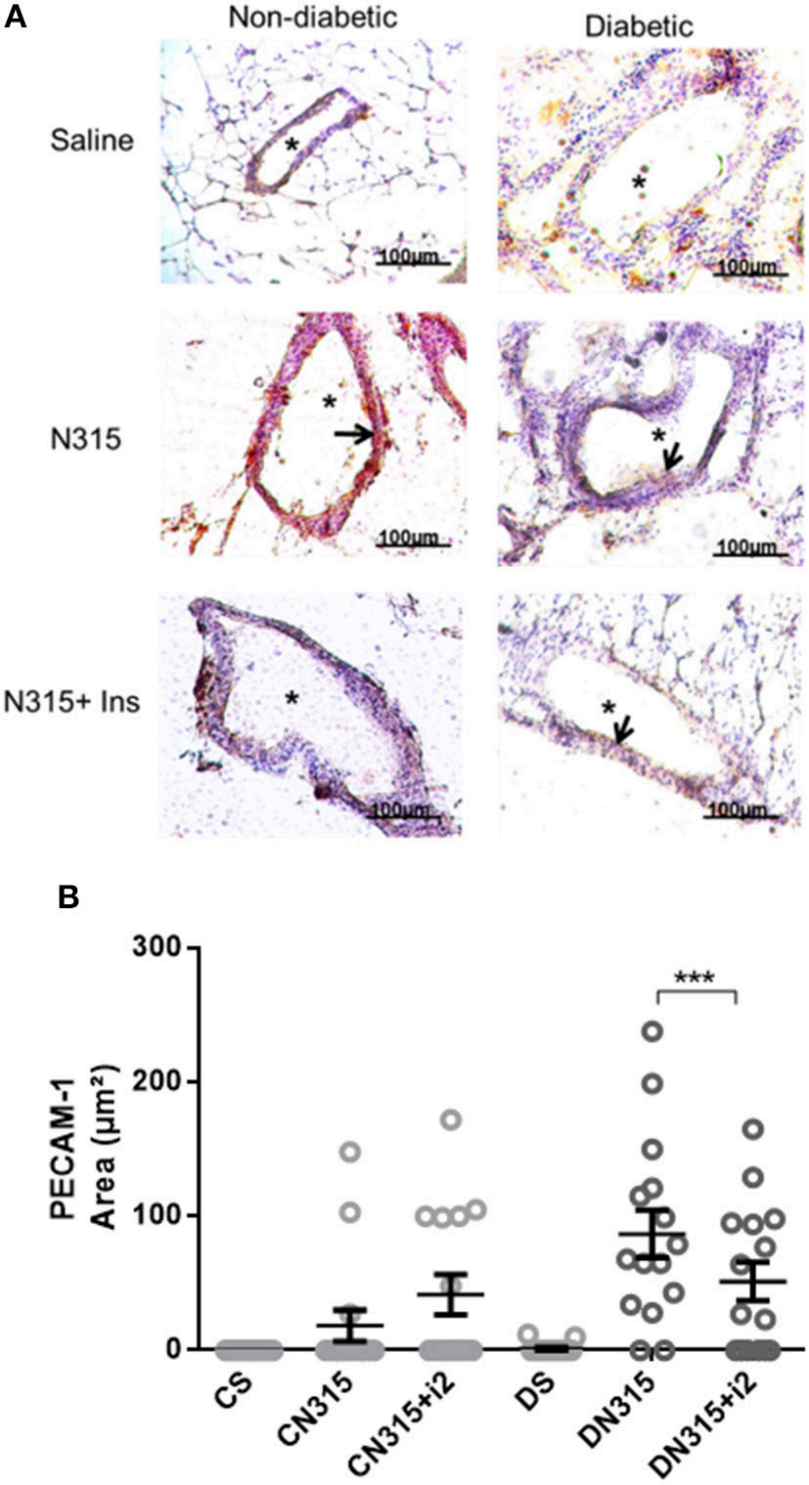
Mesenteric PECAM expression after *S. aureus* strain N315 αHL^+^ infection: role of insulin. Samples were obtained from the mesentery of diabetic or non-diabetic animals treated or not treated with insulin 3 days after *S. aureus* N315 αHL^+^ infection, and the samples were stained with haematoxylin (purple staining) and antibodies (brown staining, arrows) for PECAM **(A)**. ^*^blood vessel; bar = 50 μm. Quantification of immunostaining was performed with the software NIS-Elements AR. Values represent the mean total/diameter of the area ± s.e.m. of three animals/group (five fields were photographed per animal). **(B)** PECAM expression (μm^2^). Animals were treated with multiple doses of insulin (I2). Non-diabetic uninfected group (Cs); Non-diabetic infected group (CATCC); Non-diabetic infected; and treated with I2 insulin group (CATCC+I2); Diabetic uninfected group (Ds); Diabetic infected group (DATCC); Diabetic infected and treated with I2 insulin group (DATCC+I2). Values are shown as the mean ± SEM of 5 animals/group ^***^p < 0.001. Differences among the groups were tested with two-way analysis of variance followed by Bonferroni *post hoc* test. (GraphPad Prism version 6.0 for Windows, GraphPad Software, La Jolla, CA, USA).

Infection induced PECAM-1 expression on the mesenteric vascular endothelium of infected diabetic (D_N315_) and non-diabetic (C_N315_) rats, whereas the molecule was barely detectable in tissues from control group animals (Cs and Ds) (Figure 5A). Nevertheless, the expression in C_N315_ animals was 4-fold lower than that in D_N315_ rats (Figure 5B). Treatment with insulin maintained the PECAM-1 expression in both groups (C_N315+i2_ and D_N315+i2_). The expression of ICAM-1 and P-selectin on the vascular endothelium was not altered in any of the experimental groups (data not shown).

#### Microbicide Activity of Peritoneal Macrophages

The microbicide activity of PMs isolated from N315 αHL^+^-infected animals indicated that insulin treatment did not alter the capacity of macrophages to eliminate bacteria (mean ± SEM; CN315, 50.9 ± 3.4; CN315+i2, 48.1 ± 4.3; DN315, 46.6 ± 4.4; DN315+i2, 46.0 ± 5). These results are expressed as the percent survival.

## Discussion

The data presented here suggest that insulin modulates peritonitis induced by *S. aureu*s. Peritoneal infection caused by *S. aureus* strain ATCC 25923 in diabetic rats caused a stronger inflammation characterized by a larger number of mononuclear leukocytes and PMNs in the PeLF. Insulin treatment reduced this effect. In contrast, infection by *S. aureus* strain N315 αHL^+^ potentiated cell migration to a similar extent in both diabetic and non-diabetic rats. A single dose of insulin reduced glucose levels in non-diabetic animals but did not alter cell migration to the PeLF. Treatment with multiple doses of insulin reduced cell migration in non-diabetic animals and increased cell migration in diabetic animals.

*S. aureus* may survive phagocytosis and grow inside macrophages (30). The bacteria produce enzymes such as catalase and superoxide dismutase, which reduce the toxic effects of reactive oxygen species (ROS) and reactive nitrogen species (RNS), thus allowing bacterial survival (30, 31). *S. aureus* also may survive inside neutrophil phagosomes after phagocytosis. For example, neutrophils containing viable *S. aure*us undergo cytolysis 6 h after phagocytosis, and they are ingested by macrophages to maintain tissue homeostasis (32). It has been reported that hyperglycaemia inhibits phagocyte microbicide activity as it decreases phagocyte opsonization, reduces the granular content in neutrophils, and alters myeloperoxidase production (33, 34). In addition, isolated cells from non-diabetic animals have been shown to exhibit higher microbicide activity than those from diabetic animals (23). In contrast, in our study, cells from diabetic and non-diabetic animals displayed the same level of activity, regardless of the *S. aureus* strain that caused infection. This apparent discrepancy could result from specific bacterial virulence factors, such as cell capsule polysaccharides, which prevent phagocytosis (35). Moreover, the αHL produced by both bacterial strains analyzed causes cytolysis that reduces the phagocytic capacity of macrophages (36). In particular, the ATCC 25923 strain presented PVL, which produces pores on the leukocyte membrane, causing cytolysis (14, 37, 38). Yano et al. (39) have suggested that treatment with insulin restores the anti-*S. aureus* bactericidal activity of neutrophils isolated from diabetic animals. In the present study, insulin did not improve the microbicide activity against either of the strains analyzed. Secretion of neutrophil protease inhibitors by bacteria can block cellular activity, which may explain this outcome (40).

PMs synthetize chemokines involved in neutrophil recruitment to the site of injury (25). We evaluated the modulation of chemokine levels induced by *S. aureus* in diabetic and non-diabetic animals. Infection with the ATCC 25923 strain resulted in a higher production of CINC-1 in both diabetic and non-diabetic rats but increased the production of CINC-2 in only the diabetic animals. Treatment with multiple doses of insulin reduced CINC-1 secretion in both groups and reduced CINC-2 secretion in diabetic animals but increased it in the non-diabetic group. Infection with the N315 αHL^+^ strain resulted in higher levels of CINC-1 production in diabetic animals and non-diabetic animals. In contrast, the production of CINC-2 was not altered. Treatment with multiple doses of insulin reduced levels of CINC-1 and CINC-2 in diabetic and non-diabetic animals. These results help explain abnormalities in the course of the inflammatory response in DM, which might contribute to the increased susceptibility and severity of infections in the diabetic host as reported by several studies (23, 33, 34, 41).

In experimental studies, diabetic mice infected with Pseudomonas aeruginosa exhibit an increase in biofilms in their wounds, and insulin treatment potentialized this increase in diabetic wounds (42). In addition, infection induced by *S. aureus* is correlated with serious diseases. Multiple virulence factors can worsen the clinical profiles of patients infected by *S. aureus*. These factors may cause the variations observed during the inflammatory response to *S. aureus* (10). Another important point is that S. aureus secretes a high-affinity insulin-binding protein, which might explain why diabetic patients/animals develop insulin resistance and why normal individuals convert to diabetic patients after recurrent or longstanding infection (43). These infections in type 1 diabetic patients may increase immune dysfunction and cause the patients to become even more susceptible to different types of infections (44).

High levels of inflammatory mediators are produced locally after *S. aureus* peritoneal infection, including some essential cytokines (IL-1β, IL-6, and TNF-α) for the development of the inflammatory response (25, 45, 46). It has been reported that diabetic animals exhibit a deficient immune response, characterized by decreased secretion of IL-1β, TNF-α, and CINC-1 (22) and impairment of neutrophils and transmigration to the site of inflammation (47). In addition, we have previously shown that insulin modulates the course of lipopolysaccharide-induced acute lung inflammation, such as through affecting the production/release of pro-inflammatory (IL-1β, TNF-α) and anti-inflammatory (IL-10) cytokines as well as the protein expression of adhesion molecules (ICAM-1, E-selectin) and neutrophil migration into the lungs, depending on the availability of insulin (48). However, Pettersson et al. (49) also suggested that alloxan-treated mice show enhanced recruitment of leukocytes but impaired function. In the present study, peritoneal infection with the ATCC 25923 strain elevated the concentration of PeLF IL-1β in diabetic animals and did the same for IFN-γ in both non-diabetic and diabetic rats. Treatment with multiple doses of insulin further increased the levels of IL-1β in diabetic animals and again did the same for IFN-γ in non-diabetic and diabetic rats. A single dose of insulin reduced CINC-1 levels and cell migration to the peritoneal cavity in diabetic animals without altering the IL-1β and IL-6 concentrations. Multiple doses of insulin induced the secretion of all these cytokines. These results could also help us to understand why this infection is more serious and possibly more difficult to eradicate in the diabetic host(50–52).

Leukocyte migration into the peritoneal cavity depends on the expression of adhesion molecules on the surfaces of endothelial cells and leukocytes (53). Multiple doses of insulin affect the secretion of IL-1B and IL-6, which regulate the expression of adhesion molecules (54, 55). Thus, we analyzed the expression of P-selectin, ICAM-1, and PECAM-1 on the mesenteric vascular endothelium, and we found that non-diabetic animals infected with ATCC 25923 intensely expressed P-selectin, ICAM-1, and PECAM-1; this effect was not observed in diabetic animals. This discrepancy between leukocyte migration and the expression of adhesion molecules might be explained by the time-course of cytokine and chemokine release. Yadav et al. (56) have demonstrated the time-course and different stages of leukocyte transmigration. Following topical application of a chemoattractant such as a chemokine, an initial increase in the leukocyte rolling flux and a reduced rolling velocity are followed by a rapid leukocyte firm adhesion response. Additionally, Shibata et al. (57) have demonstrated a correlation between increased cell numbers and the total concentrations of CINC-1 and CINC-2. These authors suggested that CINC-2 and CINC-1 both play an important role in the accumulation of neutrophils during inflammation. In addition, Yano et al. (39) have suggested that treatment with insulin restores the anti-*S. aureus* bactericidal activity of neutrophils isolated from diabetic animals. In fact, insulin was not found to improve microbicide activity against either of the strains analyzed. Moreover, it was reported that soluble factors were taken into consideration, since it has been suggested that some soluble chemoattractants might bind to leukocyte surface receptors and direct PMN to the site of inflammation (58, 59). Nevertheless, we found a considerably increased inflammatory influx in diabetic animals and high levels of PeLF CINC-1. Treatment with insulin did not alter the expression of adhesion molecules, and the treatment reduced the CINC-1 concentration as well as the inflammatory influx. Indeed, the results of the present work allow us to characterize that insulin modulates important parameters that occur during peritonitis induced by *S. aureus*. Despite the fact that some results between both strains of *S. aureus* were not similar, insulin interfered with cytokine production (CINC-1, CINC-2, IFN-γ, and IL-1β), modulating the cytokines as well as the expression of PECAM-1 on the vascular endothelium, and thereby reinforcing the idea that insulin performs an important immunoregulatory effect during development of the inflammatory response.

Preclinical animal studies precede the majority of clinical trials (60). In our case, we agree that the alloxan-diabetic model has limitations. In the present study, relative to the controls, alloxan-treated diabetic rats exhibited a significant reduction in body weight gain during a 10 day period, in addition to sharply elevated blood glucose levels, and showed a significant reduction in serum insulin concentrations. Furthermore, previous studies by our group (24, 48) did not find any differences in the inflammatory response between diabetic and non-diabetic rats (10 or 30 days after alloxan injection). However, Osuchowski et al. (61) demonstrated that diabetic mice fail to organize a substantial inflammatory response to sepsis, despite the occurrence of 100% mortality, which might contribute to the increased susceptibility and severity of infections in diabetic hosts, as reported by several studies (23, 33, 34, 41). In addition, it is important to discuss how animal models contribute to enhance our understanding of the pathophysiology of not only peritonitis but also other injuries; we understand that there are individual differences and limitations of these models. Compared to the control group or a single treatment with insulin, the animals treated with more than one dose of insulin showed an increased leukocyte influx. According to Szeto et al. (62) there is need for adjustment of the insulin dosage depending on patient treatment. Inflammatory cell influx into the peritoneum is certainly the final event that depends on the production/release of pro- and anti-inflammatory mediators and activation of leukocytes and endothelial cells implicated in peritoneal inflammation induced by different *S. aureus* strains. Here, it is important to discuss the dose and timing of insulin treatment chosen in the present study and previous studies by our group (24, 28, 48), as this dose was able to restore inflammatory parameters in diabetic rats. This dose was not sufficient to return glucose levels in diabetics to normal values, but it had no effect on mortality. Thus, we believe that the effect observed in insulin-treated rats was primarily due to the increased blood levels of insulin, rather than to the reduction of glycaemia.

Infections induced by *S. aureus* are correlated with serious diseases. Multiple virulence factors can worsen the clinical profile of patients infected by *S. aureus*. These factors may cause the variations observed during the inflammatory response to *S. aureus* (10). In conclusion, the data presented show that insulin regulates the secretion of pro-inflammatory cytokines (IL-6, IL-1β, TNF-α, and IFN-γ) and, consequently, inflammatory cell influx. These results suggest that the protective effect of insulin on peritonitis could be due to the modulation of inflammatory cell migration.

## Author Contributions

PdS, SF, FN, EM, and JM conceived and designed the experiments. PdS, SF, FN, JC, FT, FC, and MS performed the experiments. PdS, SF, FN, EM, and JM analyzed the data. EM and JM contributed reagents, materials, analysis tools. SF, FN, and JM wrote the paper with the assistance of all the authors.

## Conflict of Interest Statement

The authors declare that the research was conducted in the absence of any commercial or financial relationships that could be construed as a potential conflict of interest.
